# Optimized Adaboost Support Vector Machine-Based Encryption for Securing IoT-Cloud Healthcare Data

**DOI:** 10.3390/s25030731

**Published:** 2025-01-25

**Authors:** Yoosef B. Abushark, Shabbir Hassan, Asif Irshad Khan

**Affiliations:** 1 Department of Computer Science, Faculty of Computing and Information Technology, King Abdulaziz University, Jeddah 21589, Saudi Arabia; yabusharkh@kau.edu.sa; 2 Department of Computer Science, Aligarh Muslim University, Aligarh 202001, Uttar Pradesh, India; shassan.cs@amu.ac.in

**Keywords:** machine learning, cloud computing, medical applications, healthcare data management, data encryption, data decryption, encryption time, decryption time

## Abstract

The Internet of Things (IoT) connects various medical devices that enable remote monitoring, which can improve patient outcomes and help healthcare providers deliver precise diagnoses and better service to patients. However, IoT-based healthcare management systems face significant challenges in data security, such as maintaining a triad of confidentiality, integrity, and availability (CIA) and securing data transmission. This paper proposes a novel AdaBoost support vector machine (ASVM) based on the grey wolf optimization and international data encryption algorithm (ASVM-based GWO-IDEA) to secure medical data in an IoT-enabled healthcare system. The primary objective of this work was to prevent possible cyberattacks, unauthorized access, and tampering with the security of such healthcare systems. The proposed scheme encodes the healthcare data before transmitting them, protecting them from unauthorized access and other network vulnerabilities. The scheme was implemented in Python, and its efficiency was evaluated using a Kaggle-based public healthcare dataset. The performance of the model/scheme was evaluated with existing strategies in the context of effective security parameters, such as the confidentiality rate and throughput. When using the suggested methodology, the data transmission process was improved and achieved a high throughput of 97.86%, an improved resource utilization degree of 98.45%, and a high efficiency of 93.45% during data transmission.

## 1. Introduction

In healthcare systems, making decisions necessitates the rigorous examination of large volumes of real-time data collected from sensors and wearable devices. These decisions significantly influence patient health. For instance, a smart city’s healthcare system may perform in-depth examinations on its patients and step in if they participate in behaviors that might adversely affect their health. Numerous studies have used IoT-enabled wearables to collect data that might be used to diagnose a wide range of symptoms and diseases [1]. Cloud computing (CC) has become a popular method of storing and accessing data, and digital communication has become an essential part of modern life. However, security concerns are critical in data storage and transmission [2]. Utilizing CC in the healthcare industry helps to cut down on the expenses of data storage, processing, and updating while also increasing productivity and quality. However, data security in the cloud is still inadequate, and protecting sensitive information in the cloud is a persistent problem. Therefore, cryptography is a standard component of CC technology for protecting user’s data, mainly medical data, against malicious attacks [3,4]. In light of these cryptographic challenges, a public cloud strategy was proposed for the medical cloud environment to safeguard medical data [5]. Because most applications and services have gone digital, medical services are now easily accessible over the Internet [6]. Nevertheless, security is the primary concern when providing medical services over a wireless network [7]. The key generation method locks user details and prevents such possible attacks and third-party access to the cloud system [8]. Therefore, only authorized medical professionals (users) are allowed to access the data [9].

**Figure 1:**
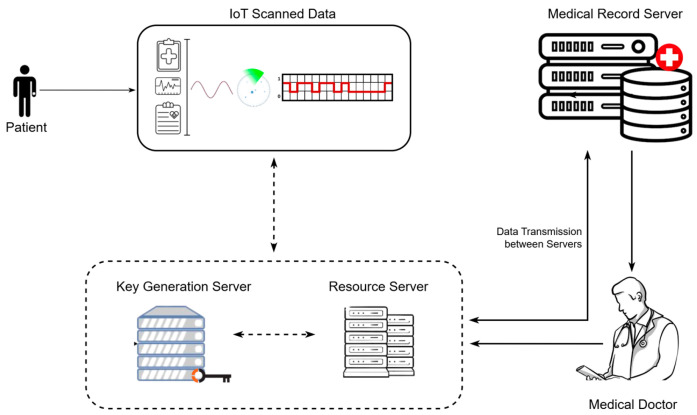
Basic cryptosystem model.

The basic working principle of a cryptosystem is shown in Figure 1. The illustration shows a medical data management and security system that uses IoT sensors to monitor patients in real time. The resource server receives sensed data from IoT devices and then stores and processes it. In order to secure data on the resource server, the key generation server transmits keys to the resource server. In order to integrate the processed data of the resource server with the patient’s current medical history, the data are relocated to the medical record server. To obtain well-informed clinical findings, including raw data from the IoT, doctors allow access to examine these records so that non-trivial diagnostic reports can be obtained.

To protect the patient’s health records from unauthorized access, the model deploys a key generation server that emphasizes how central key management and data encryption take place. The diagram shows that the model is designed to ensure data confidentiality and integrity, which are essential for preserving patient privacy and trust in healthcare systems. The key components of this model are given as follows:

IoT-sensed data: the model gathers information from a variety of IoT devices (such as wearables and sensors) that track patients’ underlying signs and symptoms, such as blood pressure and heart rate.

Resource server: Data gathered from these IoT sensors are stored and managed by this centralized server. In order to detect possible health problems, it also assists in analyzing and interpreting the data.

Key generation server: the main responsibility of this unit is to encrypt and decrypt resource server data and generate keys for secure data exchange.

Medical record server: the patient’s medical records, including past data (medical history) and IoT-processed data, are stored on this server.

Doctor: this represents the medical practitioner who has access to the resource server and medical record server to analyze data for reading history, performing diagnosis, treatment planning, and follow-up.

In cryptosystems, robust registration processes and the prevention of one-sided secret keys are vital for mitigating cyberattacks [10]. A good system also facilitates seamless communication among all cloud-associated users [11]. Hence, for cloud and associated data transmissions, numerous cryptosystems have been proposed to maintain secrecy and other privacy-related benchmarks for healthcare data [12]. However, due to the slow key generation process, such models perform best at the time of communication [13]. Consequently, attackers can forge a key that appears identical to the actual key to challenge the system’s security and steal data [14,15]. After the key is generated in the cloud storage, it is difficult to predict a detrimental event [16]. The fundamental basis for using the public cloud is to increase the privacy of the cloud environment by supplying a secret key to each individual [17,18].

The most challenging aspects of the healthcare management system are ensuring security, enhancing execution speed, and data confidentiality. In addition, the security of such a system is threatened by eavesdroppers and hackers in various ways. Several models have been developed to address these issues, such as medical electronic documentation and authorization systems and IoT-based cancer prediction [19,20]. However, these models still suffer from potential anomalies such as a high error rate, low reliability, low compatibility, high encryption and decryption times, a lack of security, and minimal throughput. Therefore, it is urgent to design an optimized encryption-based public cloud model to improve the existing healthcare management system and overcome the above-mentioned challenges. The main objective of this work was to devise a solution providing a robust cryptosystem for securing medical data against the above-mentioned attacks. In addition, the encryption method used to create the secret key for security was upgraded to include grey wolf optimization. Additionally, the present study aimed to protect medical data on a public cloud system and use international data encryption algorithm (IDEA)-based encryption.

The main highlights of this study are as follows:

▪ We developed a robust framework leveraging IoT sensors for real-time medical data collection and synchronized it with the medical record server, ensuring efficient and seamless healthcare data management.
▪ We proposed an ASVM-based GWO-IDEA approach to enhance the security of data communications in IoT-driven healthcare systems, effectively mitigating cyberthreats.
▪ We conducted comprehensive experiments to assess the model’s reliability and resilience across diverse operational scenarios.
▪ We demonstrated the superiority of the proposed method over existing techniques through extensive comparative analyses.
▪ We achieved significant enhancements in security parameters, such as encryption speed, computational efficiency, and resource utilization.
▪ We addressed key challenges in data protection and encryption, contributing to the development of secure and advanced IoT-enabled healthcare systems.

The structure of this paper is as follows: Section 2 describes relevant studies on healthcare security, while Section 3 contains descriptions of problems and concerns. In addition, the developed model is explained in Section 4, the results of the proposed technique are documented in Section 5, and conclusions regarding the proposed method are provided in Section 6.

## 2. Related Work

The following is a summary of recent studies in the literature on the security of healthcare and related systems. Chin-Ling et al. [20] offered an electronic health authorization and documentation strategy using the public key and elliptical curve (EC) encryption. Moreover, this study provides a secure healthcare system that works with both a public and private cloud background. This approach protects against various threats while allowing for unlinking, anonymity, integrity, backward security, and forward security. However, the built framework has a high error rate.

The IoT and CC are the most popular solutions to real-world challenges. Anuradha et al. [21] designed a cancer prediction system using the IoT. The cloud stores encrypted data, including cancer patients’ blood results, resulting in increased healthcare efficiency and cloud data security. However, the encryption of cancer patients’ data is time-consuming, making processing slow.

Elliptic curve (EC) cryptography and a safe framework to validate elaborate medical systems have been presented by Adesh Kumari et al. [22]. The created effective and safe framework ignores attacks and third parties. As a result, the proposed model protects the cloud against attacks and malicious activity and has a low error rate; however, it has limited reliability due to communication issues.

Cloud-based healthcare data store highly sensitive information that must be maintained securely to prevent unwanted access and protect patient information. Madhubala et al. [23] suggested an innovative hybridization data encryption technique for securing patient data in medical images. The proposed method achieved high efficiency in terms of instructional content, entropy, histogram, and other metrics. However, due to low compatibility, the attack avoidance rate was lower. An EC system was developed for testing the public system and secure cloud by Benil et al. [24]. Furthermore, medical data were encrypted, and digital signatures were produced to distribute and store them on the cloud. The duplication ensured privacy, integrity, security, and confidentiality protection from attacks and unauthorized individuals. As a result, the design can be made more straightforward.

Yang et al. [25] proposed a platform for complete health management services for the general population, which supports the growing use of intelligent sensors and intelligent health system detection technologies to expand sports health management service tools, addressing the issues with current intelligent sports health management systems’ long cycles and high costs. Muzafer et al. [26] presented a novel encryption technique based on individual block ciphering and Catalan random walks. The authors validated the suggested approach by evaluating the Catalan key generation’s time and space complexity and performing Maurer’s universal statistical test.

Abdulaziz Aldaej et al. [27] presented a study to develop a secure data exchange framework for IoT-based healthcare systems using an optimized deep learning approach. This methodology integrated particle swarm optimization with a deep neural network (PSO-DNN). This optimized network emphasizes secure transactions, efficient encryption, and healthcare diagnosis. The implementation outcomes illustrate how this strategy offers secure IoT healthcare detection. However, this study did not concentrate on patient care and provided limited efficiency.

Gia Nhu Nguyen et al. [28] developed a secure intrusion detection and data transmission strategy by integrating the efficiency of blockchain and deep belief networks (DBNs). The main aim of this study was to ensure data security in the healthcare sector against cyberthreats. Using the DBN component, this strategy identifies attacks and utilizes blockchain to securely transmit healthcare data to the cloud server. The experimental results showed that this strategy provides improved security during validation across NSL-KDD 2015, CIDDS-001, and ISIC datasets. However, this methodology lacks scalability and is less adaptable.

Prabhat Kumar et al. [29] proposed a blockchain-based DL approach for secure data transmission in IoT-enabled healthcare systems. The DL approach combines a deep sparse autoencoder (DSAE) with bidirectional long short-term memory (BiLSTM), and the blockchain utilizes an InterPlanetary File System to address the data security challenges. The validation across CICIDS-2017 and ToN-IoT demonstrated that this strategy outperforms existing methods. However, this method is computationally intensive and faces difficulty, particularly in large-scale IoT networks. Nirmala Devi Kathamuthu et al. [30] proposed a unique mechanism using the combined efficiency of deep Q-learning and neural networks to ensure privacy in IoT-based healthcare systems. This study aimed to protect the data against security threats during transmission with minimal computational overheads. The simulation results of this study demonstrated that it offered a significant reduction in cost and communication errors. However, this strategy cannot predict and mitigate unknown or emerging cyberthreats.

Monire Norouzi et al. [31] developed a hybrid approach to ensure privacy and data security in the Internet of Medical Things (IoMT). This approach combines the efficiency of the genetic algorithm with the random forest to protect patients’ medical data against cyberattacks in the IoMT environment. The implementation outcomes illustrated that this method offers safe and secure data transmission and predicts cyberattacks with high accuracy. However, this strategy lacks generalizability and also faces high computational complexity.

Xiaoying Liu et al. [32] explored a wireless-powered mobile edge computing (WP-MEC) network featuring multiple hybrid access points (HAPs) in a dynamic setting. In this network, wireless devices (WDs) harvest energy from radio frequency (RF) signals transmitted by HAPs and process their computational tasks either locally (local computing mode) or by offloading them to selected HAPs (edge computing mode). Furthermore, it emphasizes optimization techniques that minimize the long-term energy provision of the WP-MEC network while taking into account limitations related to energy, computation delay, and computation data consumption in order to pursue a green computing design. In order to adjust to the time-varying computation data and wireless channels of WDs, the transmit power of HAPs, the length of the wireless power transfer (WPT) phase, the offloading decisions of WDs, the time allocation for offloading, and the CPU frequency for local computing are all optimized in tandem. The advantages and disadvantages of the existing technique are explained in Table 1.

## 3. Problem Declaration and System Model

**Figure 2:**
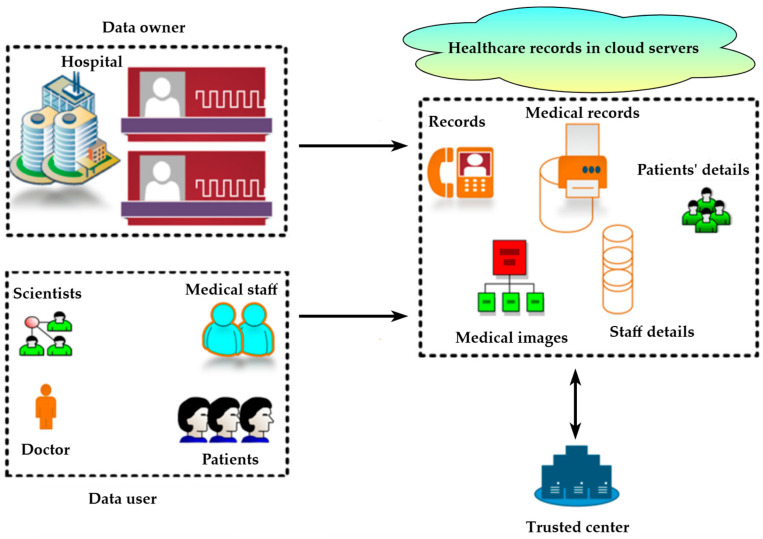
High-level view of a healthcare application system.

The utilization of CC in healthcare has expanded significantly. Throughout the epidemic, the whole healthcare system was dependent on cloud computing. In addition to physicians and nurses, all medical institutions have benefited from IT infrastructures. CC offers a contemporary solution for data collection, analysis, presentation, storage, and protection—a vital resource in businesses where data are used in practically every operation. It has caused substantial changes in various areas, including manufacturing, healthcare, and others. As a consequence, it provides several benefits to physicians undertaking healthcare functions. Furthermore, several healthcare gadgets can be employed to gather patient medical data. The system model for a healthcare application is depicted in Figure 2.

Patients, physicians, other medical professionals, and scientists are referred to as data users, whereas hospitals are referred to as data owners. Furthermore, medical records and data security are available on cloud servers (CSs). As a result, data owners are accustomed to assigning accountability and duty to specific healthcare data, as well as guaranteeing data security and data quality. Medical data are essential for treating people with severe conditions in all circumstances. As a result, data kept in cloud storage should be secure, and several models have been developed to secure them. However, they cannot protect cloud data from dangerous attacks and malicious events [33,34]. Therefore, the aim of designing this model was to develop a new, well-organized security paradigm to secure data from attacks and malicious activities. The proposed ASVM-based GWO-IDEA scheme provides medical data security, strong protection from attacks, and a high confidentiality rate, which may enhance performance.

## 4. Proposed Methodology

This paper presents a novel ASVM-GWO-IDEA for safeguarding cloud data in data transmission to shield the data owner’s medical records from intrusion and cyberattacks.

### 4.1. Medical Data Collection

Initially, medical data were gathered from an IoT-cloud-based healthcare environment. The healthcare environment included hospital centers, patients, cloud storage, and IoT devices. The patient information was collected using wearable sensors such as glucose meters, breathing sensors, accelerometers, and temperature sensors. The gathered information included the patient’s medical data, including temperature, glucose levels, blood pressure, heart rate, pulse rate, sugar level, cholesterol level, oxygen levels, etc. The gathered data were transmitted to the cloud for centralized storage. The presented study utilized the publicly available medical database named “Healthcare-IoT database [35]”. This database simulates sensor data accumulated from wearable devices in an IoT-assisted healthcare framework, and the information resembles a patient’s health information, such as blood pressure, patient ID, timestamp, sensor type and ID, heart rate, temperature, battery level, and health status. These data were transmitted to healthcare centers for remote monitoring. The database is available in CSV form on the Kaggle site, and its size is 10.86 GB.

**Figure 3:**
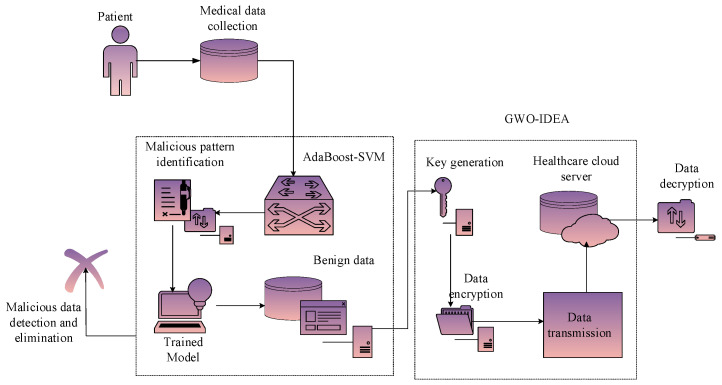
System architecture of the proposed model.

Furthermore, the medical data of data owners in hospitals were taken to evaluate the unauthorized access detection procedure. Consequently, an optimized public cloud scheme was developed to enhance the confidentiality and security of stored medical data in the cloud. As a result, the new ASVM-based GWO-IDEA model was developed in the Python environment. The proposed architecture is detailed in Figure 3.

The outcomes attained with the designed model were validated using prevailing methods to prove the efficiency of the developed process. The reason for utilizing grey wolf optimization fitness in the optimized public cloud scheme was to raise awareness of presenting attacks or malicious events at an earlier stage. Furthermore, collected IoT sensor medical data were distributed to the optimized public cloud scheme to improve the performance of healthcare data management. Consequently, the medical dataset included patient healthcare details such as heartbeat rate, blood pressure, sugar level, and body temperature deposited in the optimized public cloud scheme. Additionally, the designed method safeguards the data owner’s medical data during data communication and effectively protects the cloud-stored database.

### 4.2. Proposed Approach Terminology

There are four main components in the proposed framework: AdaBoost, SVM, grey wolf optimization (GWO), and IDEA. A brief introduction to these components is provided below.

#### 4.2.1. AdaBoost

AdaBoost, or adaptive boosting, is an ensemble learning approach employed in machine learning (ML) to solve categorization and regression problems [36]. The concept of this algorithm is training the weak classifier using the training sequence iteratively, with each successive classifier providing more weight to the data, which are incorrectly categorized. The final AdaBoost model was selected by aggregating all the weak classifiers that were utilized in the training stage along with the weight provided to them based on their classification accuracy. Typically, the weakest classifier with greater accuracy is offered with high weightage and vice versa.

#### 4.2.2. Support Vector Machine

The support vector machine (SVM) is a supervised ML approach widely deployed for performing both regression and classification tasks [36]. This algorithm is adaptable and is applied in various contexts, including image categorization, gene expression assessment, text classification, spam identification, face recognition, and anomaly prediction. This approach functions by identifying the maximum separating (optimal) hyperplane that segregates data points into various categories in the feature space. This algorithm can also handle both linear and non-linear data samples, which makes it a reliable solution for grouping data points into classes.

#### 4.2.3. Grey Wolf Optimization

GWO is a population-based meta-heuristic optimization approach developed based on the characteristics of grey wolves [37]. This approach is mathematically modeled based on the leadership hierarchy and hunting behavior (the tracking, encircling, and attacking of prey) of grey wolves to solve complex world optimization problems. The main advantage of this algorithm is its high performance in unknown cases, which improves its generalization efficiency in real-time applications. This algorithm also offers greater stability between the exploration and exploitation phases and provides a greater convergence speed. Moreover, this approach avoids local optima and enhances optimization efficiency in local searches.

#### 4.2.4. International Data Encryption Algorithm

IDEA is a symmetric key block cipher developed to offer secure data encryption. This algorithm is currently used in numerous applications, including financial transactions, secure communications, and electronic voting systems [38]. It utilizes a block cipher, which is crucial in cryptography for encrypting the data. In addition, the IDEA deploys mathematical functions such as modular arithmetic, XOR, and bit shifting to encode the original data, protecting them from external threats. The main advantage of this approach is its efficiency in terms of both software and hardware. Also, this algorithm is faster and consumes minimal processing power and memory.

### 4.3. Optimized Public Cloud Scheme

The optimized public cloud scheme [39] contains a computing service that offers public internet connectivity to third-party customers. Based on data confidentiality against the cloud server, the cloud platform has four distinct individuals. Patients, healthcare professionals, and medical organizations are referred to as users to authenticate the data owner. It also aids in evaluating and sharing healthcare data from a cloud database. In network management, the data owner examines healthcare information anytime and anywhere. The optimized public cloud framework is divided into storage and processing. Furthermore, the optimized public cloud infrastructure enables information sharing across the enterprise. Likewise, an optimized public cloud framework is also known as a self-service interface and is a safe way to transmit data.

### 4.4. Process of ASVM-Based GWO-IDEA

The proposed optimized public cloud scheme method combines the AdaBoost, SVM, grey wolf algorithm, and IDEA. At this point, the information collected about the healthcare of several patients is habitually encrypted with the help of an optimized public cloud scheme, and attacks or unauthorized access to the system are identified using the GWO fitness function. Accordingly, this research endeavored to design a robust, fast, efficient, and successful model by implementing wolf optimization with vast competence in searching and hunting the location of prey, benefitting fitness.

The developed GWO-IDEA is the better security framework for securing healthcare data; it encrypts personal information in the public cloud system. The designed model encrypts the data securely by providing the secret key of each user, and grey wolf optimization fitness continuously monitors the user details to secure the data from third parties. If the user’s secret key is not matched to the stored database, the user is denied access, and healthcare data are secured. Furthermore, GWO-IDEA encrypts the data to secure patients’ details and continuously monitors the users’ details in the auditing phase according to their hunting behavior in terms of grey wolf exploration and exploitation $Q\left(s\right)$ . The parameters used for continuous monitoring in the auditing phase are $f_{1}\left(t\right)$ and $\;f_{2}\left(t\right)$ .

- **AdaBoost SVM**

Generally, the framework uses the AdaBoost algorithm to initialize the training sample weight. The weight of each training sample builds a new decision tree with all the features. Also, it is used to boost performance in terms of accuracy, classification, and regression [38]. Moreover, AdaBoost in SVM attains better performance in SVM classification problems. It mainly focuses on the correct classification of trained samples and identifies the weak classifier using the weight of the samples. The training sample $\left(h_{t}\right)$ stored in the cloud database is initially retrieved using Equation (1).

(1) $$h_{t}=\left\{\left(a,b\right),.....\left(ai,bi\right)\right\}$$

where $\left(a,b\right)$ denotes the trained samples and $\left(ai,\;bi\right)$ indicates the number of turns in a single iteration. Then, the weight of the training samples $\left(W_{t}\right)$ is modified using Equation (2).

(2) $$W_{t}=\frac{1}{i}\mathrm{For}\;i=1.....n$$

Let i denote the training sample value and n denote the cloud database parameters. The initialization of the weight is used to identify the weak learning classification in SVM and calculate the training errors in the training samples. Thus, the training error $\left(T_{e}\right)$ is calculated using Equation (3).

(3) $$T_{e}=\sum_{N}^{i=1}W_{t},\left(ai,bi\right)\neq h_{t}\left(ai\right)$$

Furthermore, the weight of the training sample $\left(W_{t+1}^{i}\right)$ in the developed framework is updated using Equation (4).

(4) $$W_{t+1}^{i}=\frac{W_{t}\exp\left(b_{i}\times \alpha t\times h_{t}\left(ai\right)\right)}{K_{t}}$$

Let $K_{t}$ denote a normalized constant, αt denote the weight of the trained sample, and $b_{i}$ denote a binary classification. Then, based on the weight of all the trained samples, the distribution of the best weak classifier is identified, and the weak classifier error rate is determined. Finally, a weak classifier is turned into a robust classifier by updating the weights to improve the performance of the developed framework.

Moreover, SVM collects and classifies objects, information, or data. It is a non-linear mapping function used to convert the original training samples into higher dimensions. It tends to connect the input samples to high-dimensional features and identify the optimal hyperplane. The correct identification of the perfect value is used to solve the problem of overfitting and correctly classify training models. Thus, the process of SVM $\left(X\left(t\right)\right)$ in the binary classification was obtained using Equation (5).

(5) $$X\left(t\right)=\left[W_{t},\phi \left(t\right)\right]+b_{i}$$

Let $\phi \left(t\right)$ denote a high-dimensional feature space and $b_{i}$ denote a binary classification. The SVM is used to identify the features from the collected dataset. Then, these features are updated to the designed GWO-IDEA model to secure the data from third parties. The primary purpose of using AdaBoost SVM in the developed model is to enhance the classification results and minimize the overfitting issue. Moreover, the linear mapping function employed in SVM to attain high-dimension features is used to identify the perfect value.

- **Encryption and decryption using GWO-IDEA**

Several types of patient healthcare data are collected from the patients and stored in the cloud database. Moreover, a cloud database helps to keep the patient’s medical information and history safe [40]. Nonetheless, the cloud database lacks security, and the patient’s medical data can be hacked by external users, i.e., attackers and third-party applications. This problem may impact the patients’ treatment. Optimized public cloud infrastructure has been created to secure healthcare data from attacks. Additionally, an IDEA mechanism was developed based on the optimized public cloud infrastructure. To block third-party users, cloud data are spontaneously encrypted, and a key is created. Initially, the encryption is performed, and the 64-bit plaintext is shared with the 16-bit plaintext [41]. Accordingly, the designed framework converts the plaintext into a ciphertext to secure the data from third parties using bits. The IDEA transformation function process $\left(f(Y_{i}^{\prime })\right)$ is given in Equation (6).

(6) $$f(Y_{i}^{\prime })=a_{n}^{m}d_{p}⊕(a_{n}^{m}d_{p}<<z_{1}\left(1\right))⊕(a_{n}^{m}d_{p}<<z_{2}(2))⊕(a_{n}^{m}d_{p}<<z_{3}(3))⊕(a_{n}^{m}d_{p}<<z_{4}(4))$$

where $a_{n}^{m}$ denotes each common disease symptom and n represents a parameter of the stored cloud data. Additionally, $d_{p}$ represents the affected patient’s diseases, and $z_{1}\left(1\right),{\;z}_{2}\left(2\right),{\;z}_{3}\left(3\right),\;{\;z}_{4}(4)$ are a combination of some processes realized through encryption. Moreover, using the treatment serial number $\left(s_{n}^{e}\right)$ , the doctors accidentally produce cards in healthcare data that are obtained using Equation (7).

(7) $$S_{n}^{e}w^{’}\{w^{’}(w_{d}||R^{’})||{S_{n}^{e}}^{’}$$

Let $w^{’}$ denote the optimized public cloud hash function, $w_{d}$ denote a patient healthcare card, $R^{’}$ represent an accidentally nominated value, and ${S_{n}^{e}}^{’}$ denote a data owner record from the collected medical data. Affected patients are sent to the hospital for treatment. Subsequently, the doctor or physician accesses the cloud database using the secret key and starts the treatment based on the patient’s health status. Thus, the secret key is validated for encrypting private healthcare data. At this stage, numerous security problems have occurred. Thus, the actual data and collected data are validated using Equation (8).

(8) $$\alpha (A_{d})=\beta (C_{d})$$

Let α and β denote the actual comparison variables and $A_{d\;}and{\;C}_{d}$ represent the collected healthcare information from the cloud. Then, the unusual feature from the dataset is eliminated using Equation (9).

(9) $$\alpha \beta =\left\{\begin{array}{c} A_{d}*C_{d}\geq 0.2\;\;\;\;\;\;\;\;\;\;\;\;\;\;\;\;\;\;clear\;\inf o\\ otherwise\;\;\;\;\;\;\;\;\;\;\;\;\;\;\;\;\;\;\;\;\;\;\;\;0 \end{array}\right\}$$

The data owner’s medical data are secured from third parties using the optimization fitness function. At this point, the designed method continuously monitors any unauthorized access and attacks present in the cloud system for healthcare data management. As a result, the user and data owner details in the public cloud system are updated. Furthermore, the performance of the cloud server is determined using data sharing and data assessment and procedures by the users. In the auditing phase, using Equation (10), attacks and unauthorized access are monitored based on the user’s details.

(10) $$Au\left(p\right)=Q\left(s\right)+P\left(s\right)\left[\left(f_{1}\left(t\right)-s_{k})+(f_{2}\left(t\right)-s_{k}\right)\right]$$

Let $Q\left(s\right)$ represent the user-detail monitoring performance with exploitation, $P\left(s\right)$ describe the user-detail monitoring performance with exploration, and $s_{k}$ denote a security function with two parameters, $f_{1}\left(t\right)$ and $\;f_{2}\left(t\right)$ . Because $f_{1}\left(t\right)$ and $\;f_{2}\left(t\right)$ continuously monitor the grey wolf optimization fitness parameter, each user is monitored using the generated secret key ( $Se_{ky}$ ). Let us suppose that the individual user’s secret key () is not matched to the stored cloud database. In that case, neglecting whether the user’s secret key matches means contacting the user and providing secure transmission, enhancing the developed model’s security. Henceforth, the user’s non-matched secret key is updated in the security function to identify and predict the location using Equation (11).

(11) $$s_{k}=\frac{s_{k}+Q\left(s\right)}{\alpha }$$

Let α denote a unit of time. The process is repeated until the prediction of attacks and unauthorized access is complete. Moreover, the process of GWO-IDEA encryption is detailed in Algorithm 1.
**Algorithm 1** Design GWO-IDEA for encryption**begin**

**input:** Plain text/*- medical dataset of patients -*/
**output:** Ciphertext/*- encrypted information -*/
**initialize the parameters:**


*Integers* 

$Y_{1}^{’},\;Y_{2}^{’},\;Y_{3}^{’},\;Y_{4}^{’},\;a_{n}^{m},\;d_{p}$
 *and* 
r

/*- Security parameters -*/
**begin encryption:**

Update 64-bit//plain text

Shared to the GWO-IDEA

Common affected diseases of the patient z_n_(n)


Execute function of IDEA transformation using Equation (6)/*- plaintext is encrypted -*/
**cyphertext:**


z_1_(1)= z_2_(1)= z_3_(1)= z_4_(1)= … = z_n_(n)


*for(z = n; z*

≥

*n; z++)*



z_1_(1)= 
$a_{n}^{m}$
(z_1,_ z_1_(1))



*end for*

**update the parameter into the public cloud (secure storing):**

Pu_ky_/*- Puky:public key -*/Pr_ky_/*- Prky:private key -*/

Se_ky_/*- Seky:secret key -*/
**fitness of the cyphertext is measured using Equations (14) and (15)****end**


Key generation is an important part of the developed method for securing cloud data by routinely generating the key. Initially, the sender or users encrypt the patient information using the public key $Pu_{ky}$ of the receiver, even though the receiver decrypts the patient information using the private key ${\mathrm{Pr}}_{ky}$ . Additionally, the designed public cloud system generates the $Pu_{ky}$ using Equation (12).

(12) $$Pu_{ky}=Pu_{ky}*{w_{1}}^{’}$$

Let ${\mathrm{Pr}}_{ky}$ represent the private key and ${w_{1}}^{’}$ denote the collected patient information. Then, the designed public cloud system generates the secret key $Se_{ky}$ using Equation (13).

(13) $$Se_{ky}=Pu_{ky}*{\mathrm{Pr}}_{ky}*w_{1}^{’}$$

Afterward, the collected patient healthcare information is also powerfully encrypted, and keys are automatically generated to secure the data in the cloud system. At this point, the key generated and the encrypted dataset contain two cipher texts $\left(c_{t}\right)$ , $c_{1}^{\prime }\;and\;c_{2}^{\prime }$ , which are calculated using Equations (14) and (15).

(14) $$c_{1}^{\prime }=(g^{\prime }*w_{1}^{’})+Se_{ky}\to c_{t}$$

(15) $$c_{2}^{\prime }=(O_{m}+[g^{\prime }*Pu_{ky}])+Se_{ky}\to c_{t}$$

Let $O_{m}$ denote the patient healthcare information and $g^{\prime }$ be considered a number casually chosen between 1 and n. Subsequently, security is required for the encrypted healthcare information to be delivered in packets. Thus, the designed model contains security to safeguard the data from attacks. The encrypted data cannot adapt to third parties and attacks in the healthcare environment. Hence, the grey wolf optimization fitness function is used to confirm privacy by delivering proof in the data. The security and decryption processes are expressed in Algorithm 2.
**Algorithm 2** Design GWO-IDEA for decryption and security**begin**

**input:** ciphertext (encrypted data)
**output:** plaintext /*- cannot be hacked by third parties -*/
**parameter initialization:**


$f_{1}(t)$
 and 
$f_{2}(t)$
/*- security parameter -*/

select a patient 
$g^{’}$



Gather 
$O_{m}$
 from the cloud server /*- 
$O_{m}$
 healthcare information -*/
**update grey wolf optimization:**


*co-begin*


  **Au(p) = Q(s) + P(s) [**
$\left(f_{1}\left(t\right)-S_{k})+(f_{2}\left(t\right)-S_{k}\right)$
**]**/*- auditing phase for monitoring user details -*/


*co-end*


**predict the position:**/*- using Equation (11) -*/


*co-begin*


  

$S_{k}=\frac{{Q(s)+S}_{k}}{\alpha }$

/*- 
α
 unit time -*/


*co-end*


**data transmission:**

/*- source to destination -*/


/*-
P(s)
 performance of exploration -*/


/*-
Q(s)
 performance of exploitation -*/

**stored data from the cloud** 
$c_{d}$

**objective function** 
${se}_{ky}$



*if (T[*

$C_{d}$

*] > T[*

${Se}_{ky}$

*])*



   conditions satisfied



*else*



   process repeated// security level verification


*end if*




*if*

$\left(D_{t}\leq s(t)\right)$

/*- s(t) = short duration -*/

   healthcare data of patient extent at the doctor



*else*



   not disturb/*- data communication -*/


*end if*


**start decryption:**


*convert ciphertext into plain text*




*evaluating the encrypted key*




*best solution*

**end**

Initially, a big IoT sensor collects healthcare information from the patient’s body. This can include many illnesses and parameters such as blood pressure and heart rate. Additionally, the gathered healthcare information is stored in the cloud database to secure and manage the data. Consequently, several patients’ healthcare information can be hacked during data communication by attacks and third parties. Thus, an encryption technique is used to secure the information during delivery. Moreover, the fitness function of a grey wolf is initiated in the security level verification system. Then, an attack is introduced, using the designed method to check its efficiency. Therefore, while healthcare information is not disturbed during data communication, third parties and attackers cannot start to decrypt it, and the original text is provided to legitimate users.

## 5. Results and Discussion

In medical applications, cloud computation is commonly used to make healthcare information accessible from anywhere in the world. Moreover, to enhance the security performance of healthcare systems, an ASVM-based GWO-IDEA technique was designed, and the developed model was executed in Python Library Keras, using the public cloud ClearDATA. Furthermore, the medical healthcare dataset was encrypted and securely stored in the designed cloud system. The user accessed the stored information using the secret key. The parameters used for the continuous monitoring of user details are $f_{1}\left(t\right)$ and $f_{2}\left(t\right)$ . The initial iteration count was set at 500, and the designed model attained the best fitness value of 99.55 for 500 iterations and the worst fitness value of 75.64 for 50 iterations. This demonstrates that, at each iteration, the designed model updates itself and enhances its own outcomes. The outcomes obtained with the developed technique were validated using prevailing models. Security experiments were conducted to evaluate the resilience of the proposed IoT-assisted healthcare system under various cyberthreats. The experimental design consisted of two phases: before the attack (BA) and after the attack (AA). In the BA phase, medical data were securely transmitted between devices after encryption using the proposed algorithm. In the AA phase, different security attacks were introduced to assess the system’s robustness. The study implemented several cyberattacks, including DoS and DDoS, by flooding the system with requests to overwhelm its resources. Key parameters such as the request rate, attack duration, and resource consumption were carefully measured. BF attacks were carried out using automated scripts to crack authentication credentials, analyzing factors such as password complexity, the number of attempts, and system response time. MITM attacks were simulated by intercepting and modifying transmitted data, with evaluations focusing on the encryption strength, data tampering success rate, and detection time. The system’s overall performance was assessed using critical security metrics. Data confidentiality was measured by evaluating the encryption effectiveness, data integrity was assessed to ensure that the transmitted information remained unaltered, and the processing time (running time) was measured to help gauge the computational impact of the security mechanisms. Through these experiments, this study validated the proposed model’s ability to enhance security and mitigate major cyberthreats in IoT-assisted healthcare environments.

### 5.1. Case Study

**Figure 4:**
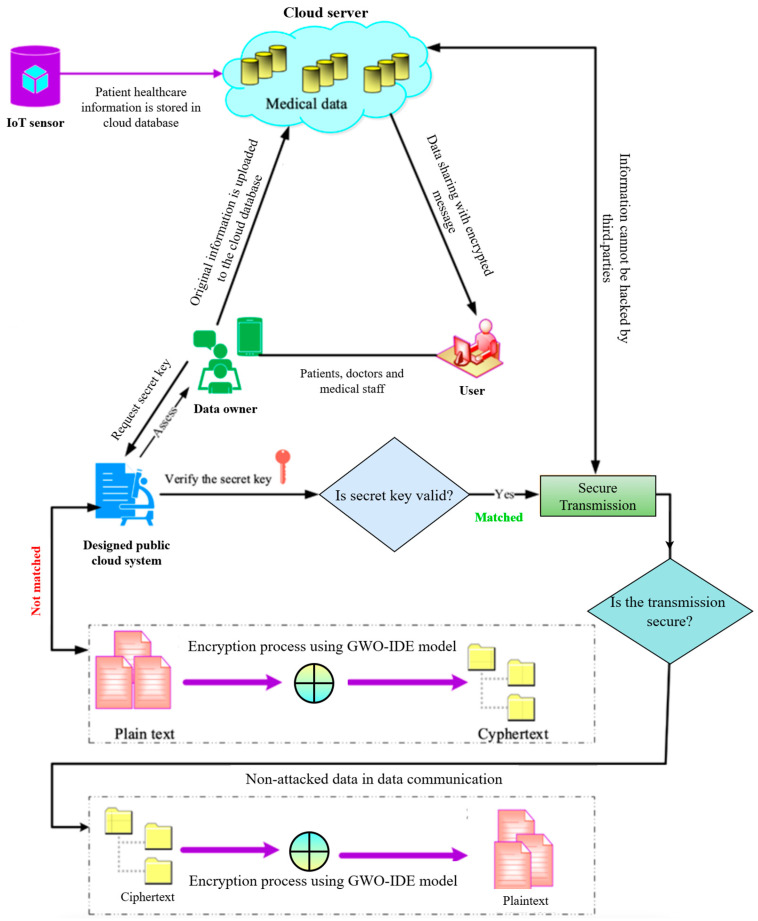
Workflow of the proposed framework.

Let us consider that a large amount of information in the form of big IoT sensor healthcare data is collected and stored in a public cloud database. Several big IoT sensors can be inserted into the human body to monitor health parameters such as heart rate and body temperature, and the sensors’ datasets are stored in the system and uploaded to the cloud using the Internet. Consequently, these data can be monitored anywhere throughout the Internet. The overall performance of the proposed technique is illustrated in Figure 4. The designed cloud system monitors users’ details and behavior continuously using grey wolf optimization fitness in the trusted center. The initial step of the developed model is data sharing, which shares the data with the data owners and requires user IDs and passwords to access the system. Then, the medical healthcare data are compressed and trained into the system. Hereafter, the encryption process is started, which sends the secret key and stores the data in the cloud system. If the secret key is not matched, the process is repeated until a suitable secret key has been authenticated.

### 5.2. Security Performance Analysis

The security performance of the developed model was evaluated by comparing the performance of the system in two cases: before the attack (BA) and after the attack (AA). In the BA case, the medical data/files were transmitted between devices in an IoT-assisted healthcare system after encrypting them using the proposed algorithm. In the AA instance, several security threats such as denial of service (DoS), distributed DoS (DDoS), brute force attacks (BF), and man-in-the-middle attacks (MITM) were launched through data packets to validate how the proposed system protected the data from potential threats. Here, metrics such as data confidentiality, data integrity, and overall processing time (running time (RT)) were assessed in both instances.

**Figure 5:**
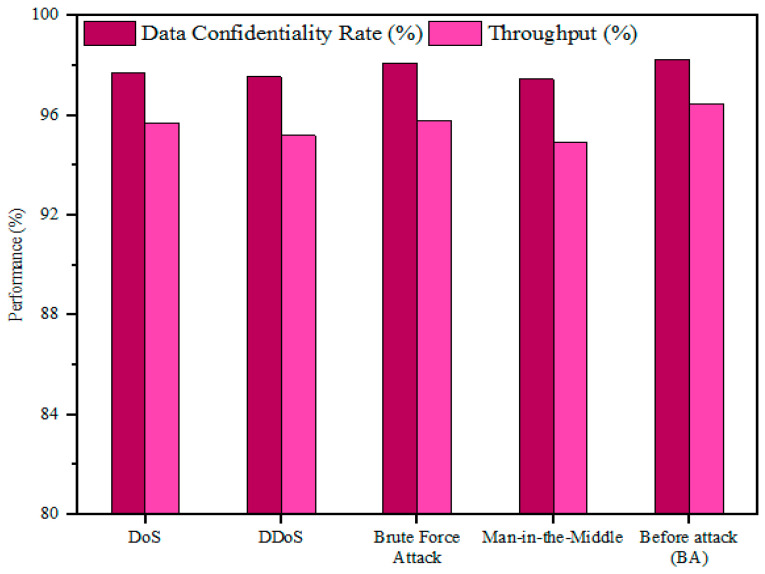
Analysis of throughput and data confidentiality rate.

**Figure 6:**
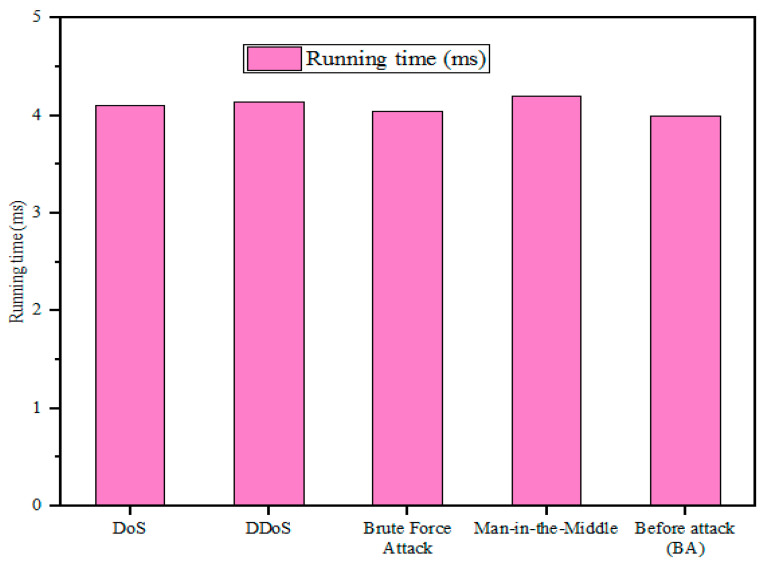
Assessment of running time against security threats.

The developed model’s performance against different security threats is analyzed in Table 2 and Figure 5 and Figure 6. Before the attacks were launched, the developed model had a data confidentiality rate of 98.25%, a throughput of 96.45%, and a running time of 4 ms. However, these parameters were reduced after the above-mentioned attacks were executed. After the DoS attack was launched, the designed strategy showed a confidentiality rate of 97.70%, a throughput of 95.70%, and a running time of 4.10 ms. Similarly, for the DDoS case, the presented framework achieved a data confidentiality rate of 97.55%, a throughput of 95.20%, and a running time of 4.14 ms, while for the brute force attack, the data confidentiality rate and throughput were reduced to 98.10% and 95.80%, respectively, and the running time was increased to 4.05 ms. Finally, after the man-in-the-middle attack case, the system showed a throughput of 96.45%, a data confidentiality rate of 97.45%, and a running time of 4.2 ms. From the analysis, it was observed that the performance deviation between BA and AA cases is very small and negligible, which demonstrates that the proposed strategy offers robust protection against vulnerable security threats.

In addition, it can be observed that the designed approach showed greater protection against brute force attacks compared to other threats. This assessment demonstrates that the proposed methodology is robust and scalable in aiding protection against security threats.

### 5.3. Performance Evaluation

The ASVM-based GWO-IDEA technique was assessed by comparing its performance metrics with those of other prevailing methods. Consequently, the data sharing and secure authentication (DSSA) technique [42], and secure data transmission (SDT) model [43] were validated with a designed method using metrics, such as confidentiality, running time, and resource utilization. Subsequently, the experimental results for the proposed strategy were validated through a detailed comparison with the above-mentioned models.

#### 5.3.1. Encryption Time (ET)

**Figure 7:**
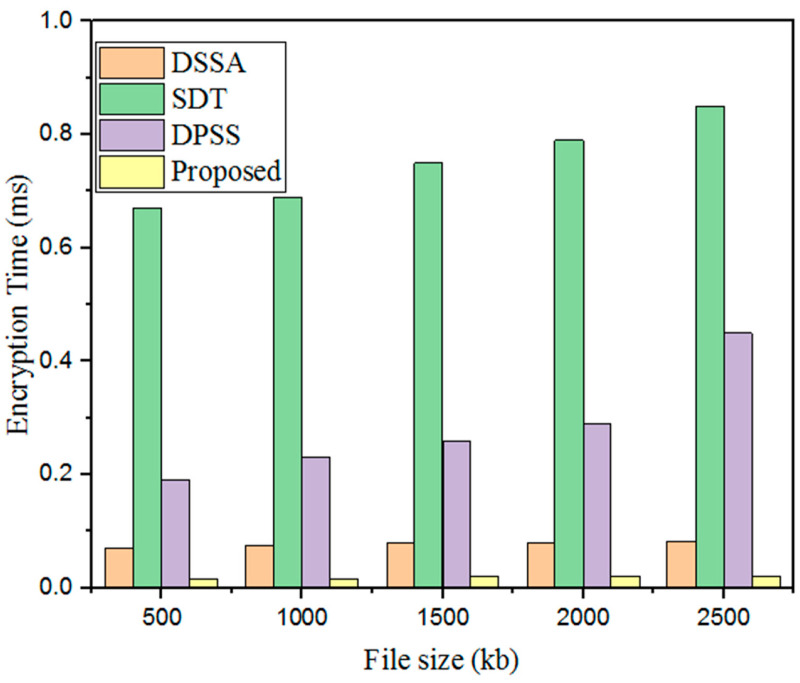
Encryption time comparison with the proposed and existing models.

Generally, the ET refers to the total required time for the conversion of plaintext into ciphertext; a lower ET indicates better performance. Also, the security of the developed technique was enhanced by key generation. Moreover, the encryption time of the designed GWO-IDEA technique was validated using existing models such as DSSA, SDT, and DPSS, as shown in Figure 7 and Table 3.

Here, the results regarding the developed technique’s ET were validated using prevailing methods. The existing methods showed high ETs for a 500 kb file: DSSA at 0.07 ms; SDT at 0.67 ms; and the DPSS model at 0.19 ms. In addition, the designed GWO-IDEA achieved an encryption time of 0.014 ms.

#### 5.3.2. Resource Utilization

The essential resources for completing the whole method were assessed using the utilization of resources for the designed method. The resources utilized included storage space, charge, time spent on the work, and execution time. The resource utilization was calculated using Equation (16).

(16) $$R_{u}=\frac{A\left(WH\right)}{T\left(AH\right)}$$

**Figure 8:**
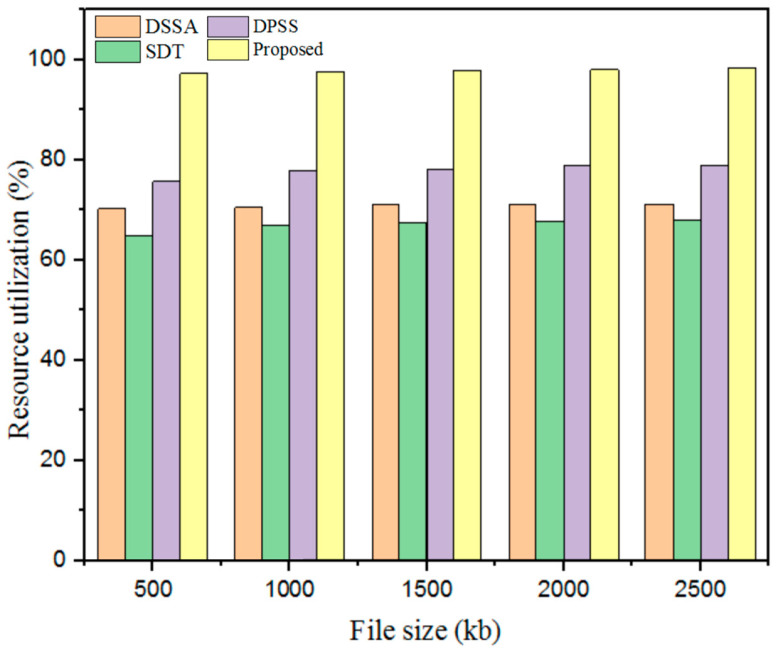
Resource utilization comparison with the proposed and existing models.

Let $A\left(WH\right)$ represent the actual number of working hours for the resources and $T\left(AH\right)$ denote the total available hours. Furthermore, the resource utilization of the designed GWO-IDEA technique was validated using existing models such as DSSA, SDT, and DPSS, as described in Figure 8 and Table 4.

Therefore, the outcomes obtained with a developed technique in terms of resource utilization were validated using prevailing methods. For a 500 kb file, the existing methods showed less resource utilization: the DSSA method at 70.2%; the SDT technique at 65%; and the DPSS model at 75.67%. The designed GWO-IDEA achieved 97.23% resource utilization.

#### 5.3.3. Throughput

**Figure 9:**
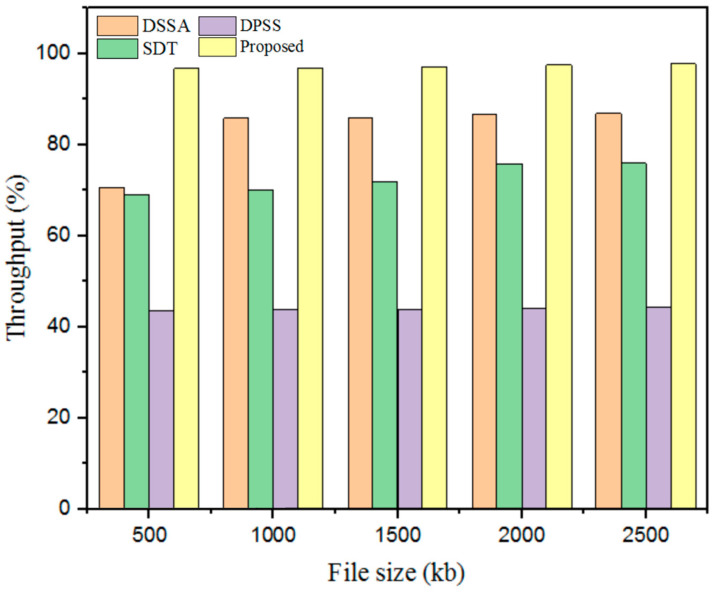
Throughput comparison with the proposed and existing models.

Throughput is the most vital parameter in CC for assessing and securing data during data communication. Furthermore, throughput differs depending on the proposed model’s run time. Subsequently, the outcomes obtained with the designed technique were compared with those obtained with other existing techniques, as detailed in Figure 9 and Table 5.

Therefore, the outcomes gained from the developed technique’s throughput were validated with prevailing methods. The existing methods, such as the DSSA method, attained less throughput at 70.67%, the SDT technique achieved 69%, and the DPSS model gained 43.6% for a 500 kb file size. In addition, the designed GWO-IDEA achieved 96.78% throughput while comparing other models’ designed techniques to attain better throughput.

#### 5.3.4. Decryption Time (DT)

**Figure 10:**
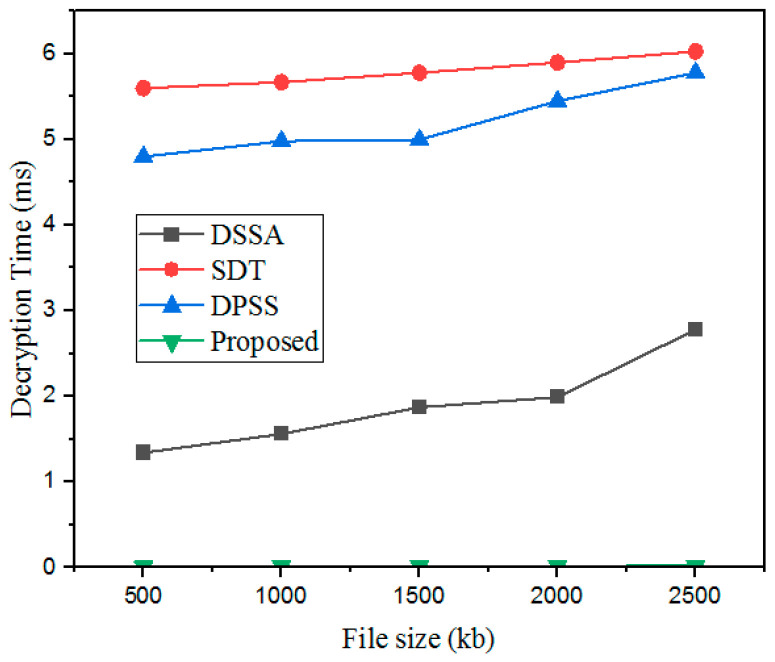
DT comparison with the proposed and existing models.

DT is commonly referred to as the total time required to convert ciphertext into plaintext. It is the reverse process of ET. Moreover, the decryption time of the designed GWO-IDEA technique was validated with existing models such as DSSA, SDT, and DPSS, as described in Figure 10 and Table 6.

The DT outcomes obtained with the developed technique were validated using prevailing methods. For a 500 kb file, the existing methods showed high DTs: the DSSA method took 1.34 ms; the SDT technique took 5.6 ms; and the DPSS model took 4.8 ms. In addition, the designed GWO-IDEA achieved a 0.01 ms decryption time.

#### 5.3.5. Running Time (RT)

**Figure 11:**
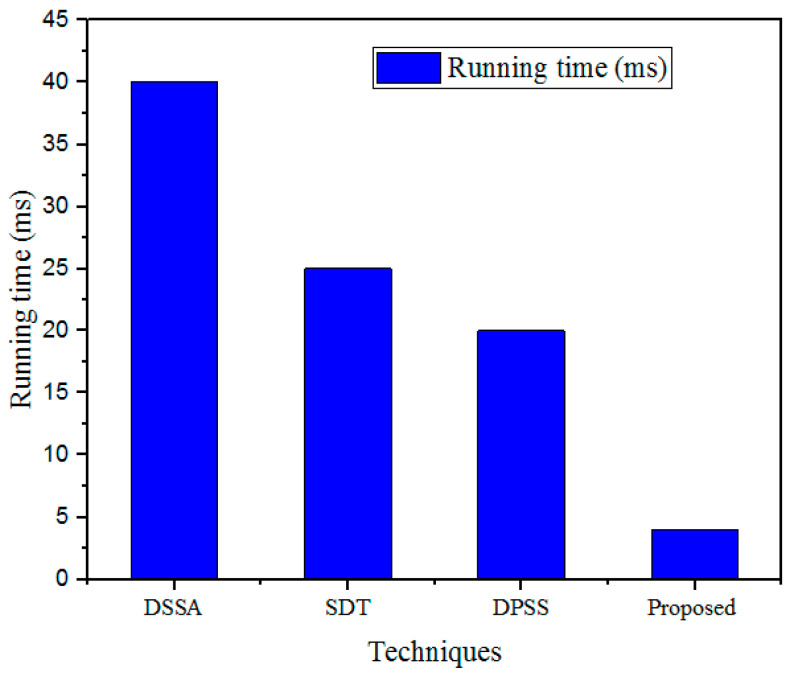
Comparison of RTs.

The RT is an essential parameter in this research; a method with a lower RT is more effective than that with a higher RT. The running time of the designed GWO-IDEA technique was validated using existing models such as DSSA, SDT, and DPSS, as described in Figure 11 and Table 7.

The RT outcomes obtained with the developed technique were validated using prevailing methods. For a 500 kb file, the existing methods showed high RTs: the DSSA method took 40 ms; the SDT technique took 25 ms; and the DPSS model took 20 ms. In addition, the designed GWO-IDEA achieved a 4 ms running time.

#### 5.3.6. Efficiency

**Figure 12:**
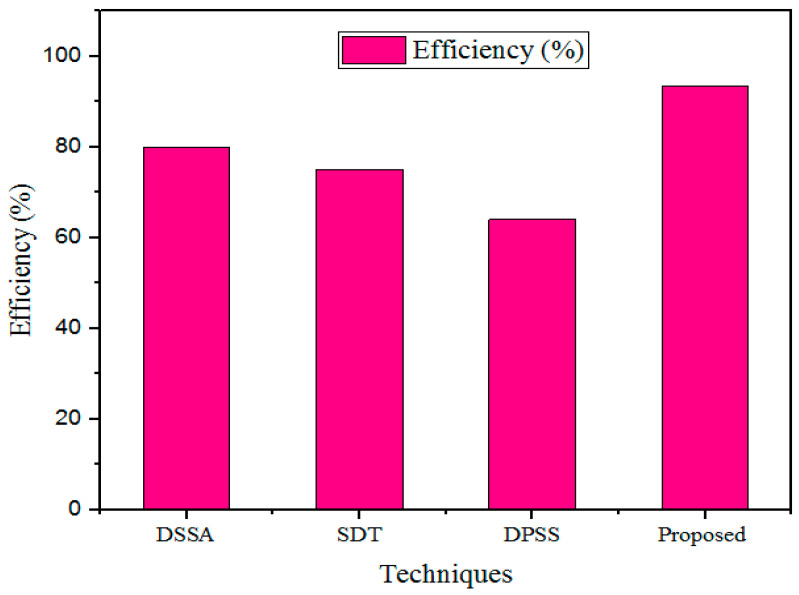
Comparison of efficiency.

The security performance of the medical healthcare system was considered efficient, and the proposed method’s overall performance was proved by measuring the communication overheads. The efficiency of the designed GWO-IDEA technique was validated using existing models, such as DSSA, SDT, and DPSS, as described in Figure 12 and Table 8.

The efficiency outcomes obtained with the developed technique were validated using prevailing methods. For a 500 kb file, the existing methods showed lower efficiency: the DSSA method had 80% efficiency; the SDT technique had 75% efficiency; and the DPSS model had 64% efficiency. In addition, the designed GWO-IDEA achieved 93.45% efficiency.

#### 5.3.7. Confidentiality

**Figure 13:**
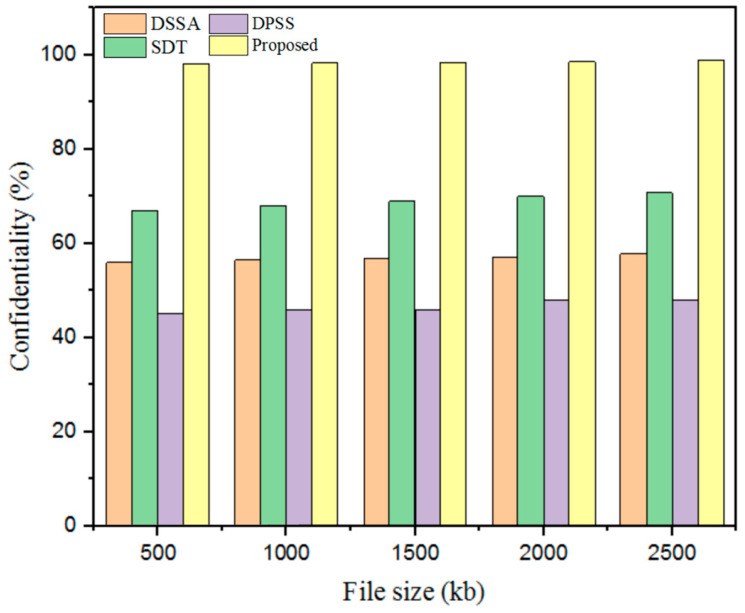
Confidentiality comparison.

Confidentiality generally applies to the original information and established information obtained during data communication. The confidentiality results obtained for the proposed technique in terms of parameters such as DSSA, SDT, and DPSS are described in Figure 13 and Table 9.

The confidentiality outcomes obtained with the developed technique were validated using prevailing methods. For a 500 kb file, the existing methods showed lower confidentiality: the DSSA method had 56% confidentiality; the SDT technique had 67% confidentiality; and the DPSS model had 45% confidentiality. In addition, the designed GWO-IDEA achieved 98.25 confidentiality.

### 5.4. Comparison with Hybrid Models

In this section, the proposed model’s performance is compared with that of recently developed hybrid models such as the particle swarm optimization-based deep neural network (PSObDNN) [27], blockchain with the deep belief network (BC-DBN) [28], the blockchain-orchestrated deep sparse autoencoder with bidirectional long short-term memory (BCoDSA-BiLSTM) [29], the deep Q-learning-based neural network (DQbNN) [30], and genetic algorithm-based random forest (GA-RF) [31]. The parameters used for the comparative assessment included encryption and decryption time, throughput, data confidentiality, resource utilization rate, system efficiency, and running time.

**Figure 14:**
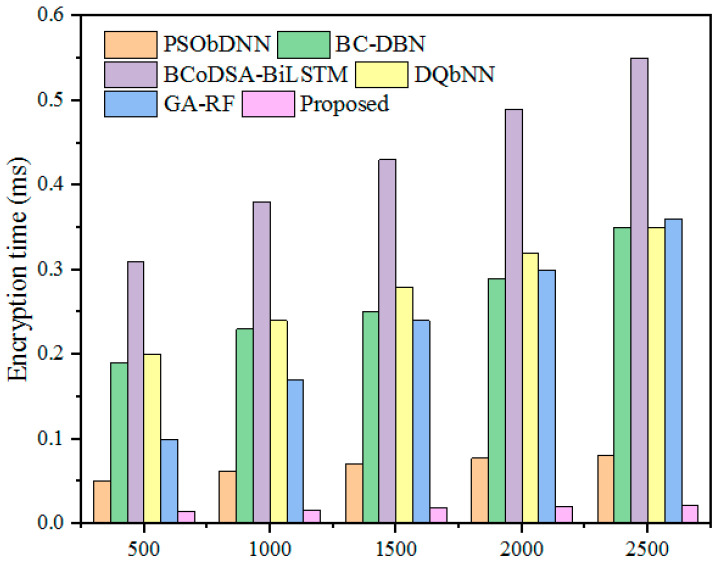
ET comparison with hybrid models.

The ET metric measures how the model converts the original data into an encoded form. Here, this parameter was assessed by increasing the input file size from 500 to 2500 kb. For a 1500 kb file, the above-mentioned hybrid models, namely PSObDNN, BC-DBN, BCoDSA-BiLSTM, DQbNN, and GA-RF, obtained encryption times of 0.071, 0.25, 0.43, 0.28, and 0.24 ms, respectively. On the other hand, the designed methodology obtained a minimum data encryption time of 0.09 ms for a 1500 kb file. The reduced ET indicates that the presented algorithm encrypts the input data into another form quickly and efficiently. Figure 14 displays the ET comparison with hybrid models, and Table 10 tabulates the ETs.

**Figure 15:**
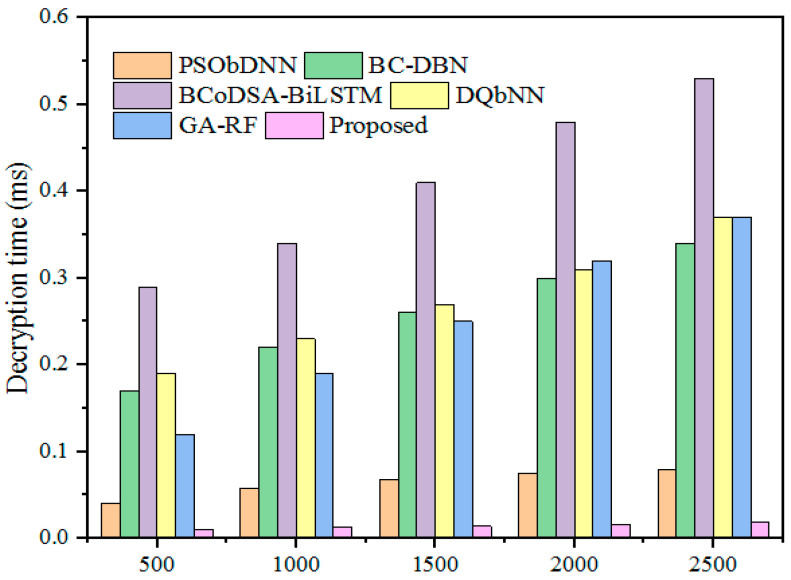
DT comparison with hybrid models.

The DT is the time taken by the model to decode the encrypted files. Figure 15 shows the DT comparison. The above-mentioned existing techniques obtained decryption times of 0.067, 0.26, 0.41, 0.27, and 0.25, respectively, while the proposed methodology consumed a minimal decryption time of 0.014 ms for decoding a 1500 kb file. This reduction in decryption time highlights that the designed approach can efficiently decode the encrypted data quickly compared to existing models. Table 11 presents the DT comparison with different hybrid models across varying file sizes.

**Figure 16:**
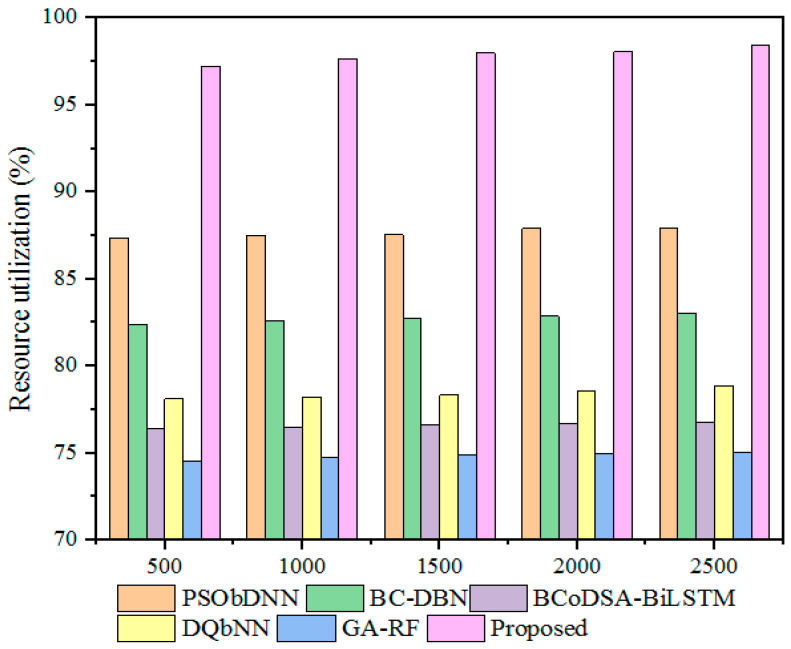
Resource utilization rate comparison with hybrid models.

The resource utilization rate measures how effectively the system uses the available resources in the IoT network during its operation. This metric also illustrates the model’s efficiency in reducing energy consumption, energy wastage, etc., and it is measured in terms of percentage (%). Figure 16 displays a comparative evaluation of the resource utilization rate of the proposed method with the corresponding rates of different hybrid models. The conventional hybrid models, including PSObDNN, BC-DBN, BCoDSA-BiLSTM, DQbNN, and GA-RF, achieved resource utilization rates of 87.54%, 82.73%, 76.65%, 78.34%, and 74.86%, respectively, while the designed approach achieved a resource utilization rate of 97.99% for a 1500 kb file size. The improved resource utilization for the developed model shows that it effectively utilizes the available resources in the network. Table 12 tabulates the resource utilization comparison with different hybrid models for varying file sizes.

**Figure 17:**
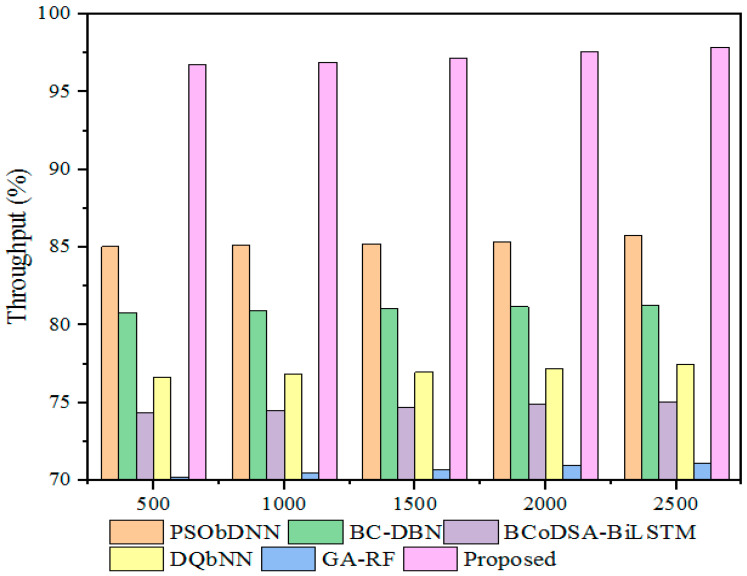
Throughput comparison with hybrid models.

Throughput measures the model’s effectiveness by successfully transmitting data from one device to another. Figure 17 displays the throughput comparison. The existing hybrid models PSObDNN, BC-DBN, BCoDSA-BiLSTM, DQbNN, and GA-RF achieved throughputs of 85.22%, 81.06%, 74.71%, 76.98%, and 70.72%, respectively, while the designed strategy obtained 97.21% for a 1500 kb file size. The increase in throughput for the proposed method demonstrates that it successfully transmits data between the devices in healthcare centers. Table 13 tabulates the throughput comparison with existing hybrid models for varying file sizes.

**Figure 18:**
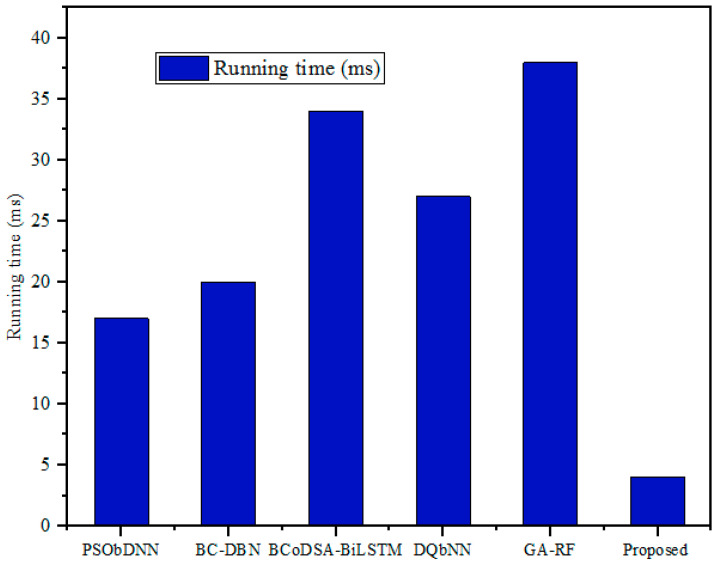
Running time validation with different models.

The running time denotes the total time consumed by the system for performing tasks such as file encryption, decryption, data processing, and transmission. The conventional hybrid models PSObDNN, BC-DBN, BCoDSA-BiLSTM, DQbNN, and GA-RF obtained running times of 17, 20, 34, 27, and 38 ms, respectively. The designed strategy consumed a minimal running time of 4 ms, which highlights its computational efficiency and capacity to perform tasks quickly. Table 14 and Figure 18 present the running time comparison with hybrid models.

**Figure 19:**
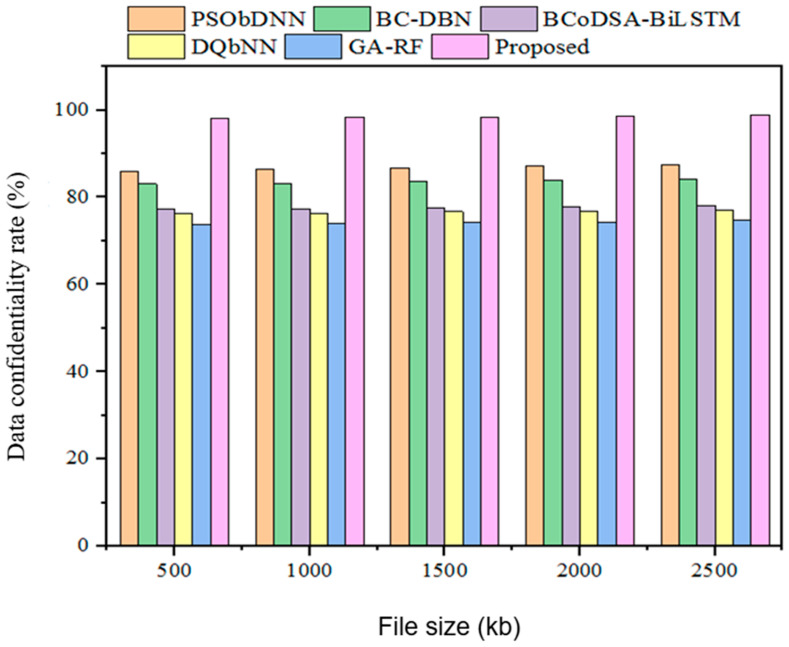
Data confidentiality rate comparison with hybrid models.

Subsequently, the data confidentiality rate was assessed to validate how the model provides security to the IoT-based healthcare system and protects against vulnerabilities. Figure 19 and Table 15 present the comparative assessment of data confidentiality. The above-mentioned existing hybrid models obtained data confidentiality rates of 86.7%, 83.68%, 77.58%, 76.72%, and 74.24%, respectively, while the proposed method achieved a 98.49% data confidentiality rate for a 1500 kb file size. The enhancement of the data confidentiality rate demonstrates that the designed methodology provides significant protection for medical information against malicious threats and other vulnerabilities.

**Figure 20:**
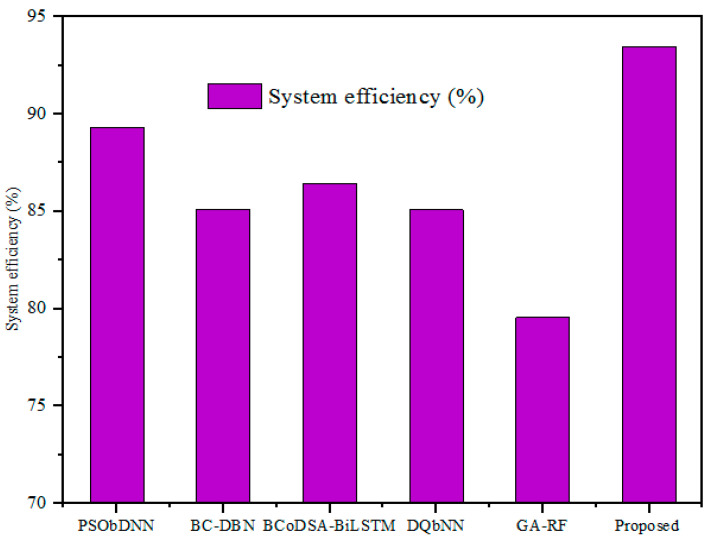
System efficiency comparison.

The system’s efficiency denotes the capacity of the proposed model to enhance its own performance in terms of metrics such as resource utilization, confidential time, and computational overheads. Figure 20 and Table 16 illustrate a comparison of system efficiency with hybrid models. The proposed strategy obtained a system efficiency of 93.45%, while the conventional hybrid models showed system efficiencies of 89.32%, 85.10%, 86.43%, 85.09%, and 79.56%, respectively. This comparative assessment demonstrates that the designed model has the potential to improve performance in IoT-based healthcare.

The comprehensive comparative assessment highlights that the designed strategy outperformed the existing models in terms of the data confidentiality rate, resource utilization, system efficiency, and throughput. In addition, the developed framework showed significant reductions in metrics of computational overheads (running time, encryption time, and decryption time).

### 5.5. Discussion

The presented work has developed a novel strategy to ensure the security of medical data during transmission in an IoT-cloud-based healthcare environment. The developed model integrates the benefits of machine learning, meta-heuristic optimization, and encryption algorithms to protect data against cyberattacks through efficient encryption. The novelty of this work lies in its integration of AdaBoost, SVM, GWO, and IDEA into a single scheme for optimized data encryption. Machine learning components perform attack detection to ensure that only benign data are transmitted in the healthcare system. After attack detection, the malicious data are removed, and only benign data are entered into the encryption block in which GWO-IDEA was developed. This algorithm encodes the original data and converts them into another form that cannot be decoded or accessed by third parties. Finally, the encrypted data are transmitted through an unsecured communication medium, and the received file is decrypted at the receiver end. The proposed framework was modeled in Python, and it was validated using public IoT healthcare data from the Kaggle site. The outcomes of its implementation in terms of confidentiality, throughput, encryption and decryption times, running time, resource utilization rate, and system efficiency were assessed. Also, a detailed comparative assessment with existing strategies was conducted, which highlighted that the designed mechanism provides superior performance in terms of metrics such as throughput, confidentiality, resource utilization, and system efficiency. Additionally, the presented framework resulted in reductions in running time, encryption time, and decryption time, which demonstrates its potential to handle computational overheads in a real-time environment. This comparative assessment illustrates that this proposed combination performs better than existing optimization models and ensemble learning approaches, making it a reliable solution for ensuring security in a healthcare environment. The complete performance of the proposed technique is detailed in Table 17.

In addition, it was observed that the utilization of GWO significantly reduced the computational requirements, thereby minimizing the implementation cost. Furthermore, the security performance assessment in the before-attack and after-attack cases illustrates that the proposed algorithm offers greater protection against unknown security threats, such as DoS, DDoS, BF, and man-in-the-middle attacks. Although the proposed strategy offered improved outcomes, certain challenges need to be addressed for optimal performance in real-time healthcare systems. Firstly, the designed strategy lacked interpretability, making it difficult for professionals to understand the concept and workings of the framework in threat detection. Secondly, the proposed work did not concentrate on latency issues that are crucial in the healthcare environment for efficient decision-making. Thirdly, this work did not consider the sensing errors created by IoT devices that may adversely affect performance and patient health. Although the system’s performance was assessed for increasing file sizes, its adaptability across diverse healthcare scenarios or datasets was not validated, which limits its applicability in real-time systems. It is to be noted that the computational cost increases when AdaBoost and SVM are combined. While SVM uses quadratic programming to solve a classification problem, AdaBoost uses iterative reweighting and weak classifier training. The classification pipeline’s integration of encryption processes adds another level of complexity, which lengthens the processing time. Furthermore, scalability issues have been reported; IoT devices frequently produce huge amounts of healthcare data, including data from real-time patient monitoring devices. AdaBoost–SVM-based encryption could be difficult to scale in order to meet the requirements for saving these mass data.

## 6. Conclusions

The IoT is an important component of the rapidly advancing automation observed in the healthcare industry. The subfield concerned with medical research is frequently referred to as the Healthcare Internet of Things (H-IoT). Data collection and processing are at the core of the H-IoT application. The incorporation of ML algorithms into the H-IoT is an absolute need in light of the mass data associated with healthcare industry predictions. Data privacy and security are the primary concerns in the cloud environment. In this study, a novel AdaBoost SVM-based GWO-IDEA was developed to address the challenges of cloud database security. The suggested approach increases the system’s reliability by achieving a balanced tradeoff between the security- and privacy-related parameters of mass medical data. Moreover, known metrics such as the RT, confidentiality, ET, and DT were used to estimate and compare the performance of our method against state-of-the-art approaches. The suggested model performed better than prior techniques, with a better ET of 0.014 ms, DT of 0.01 ms, and processing time of 4 ms. Furthermore, the developed model achieved 97.86% throughput, 98.45% resource utilization, and 98.98% confidentiality. Additionally, it achieved a bottleneck efficiency of 93.45%, often improving the security of the healthcare management system by encrypting the data and securely storing them in the public cloud system using secret keys. Furthermore, the experimental results were improved using two fitness functions that yielded minimal ET and DT. In the future, we will study hybrid optimization with IDEA to improve the security of such a system that allows role-based access to authorized users. This may address patients’ privacy issues impacting their confidence and desire to use such applications (apps). Another possible refinement may be the amalgamation of deep reinforcement learning (DRL) techniques for adaptive learning capabilities and the potential for dynamic resource allocation, which will be promising for mitigating the above-mentioned challenges while providing a robust benchmark for evaluating the performance of the proposed method.

## Figures and Tables

**Table 1:** Overview of the existing techniques.

Author	Technique	Advantages	Disadvantages
Chin-Ling Chen et al. [20]	An innovative system for authorizing medical records electronically	Anonymity Integrity Provides unlinkability Backward and forward security	High error rate Large data size
Anuradha et al. [21]	Design of a cancer prediction scheme using the IoT	Enhances healthcare performance Better security for cloud data	Long encryption time High attack rate
Adesh Kumari et al. [22]	EC cryptography and a secure structure	Malicious activity prevention is high due to securing cloud data Lower error rate	Lower reliability
Denis and Madhubala [23]	Novel hybridization encryption data technique	Entropy is high Better histogram	Less attack prevention Lower compatibility
Benil et al. [24]	EC system	Guaranteed safeguarding of security, privacy, integrity, and confidential data from attacks and third parties	Design complexity
Yang et al. [25]	Edge computing and artificial intelligence	High resource utilization Intelligent data management	Highly expensive High computational time
Muzafer et al. [26]	Block ciphering using Catalan random walks	Ensures security of multimedia content	High data-processing time High error rate
Abdulaziz Aldaej et al. [27]	Particle swarm optimization with a deep neural network	Secure transactions Healthcare diagnosis	Not concentrated on patient care and limited efficiency
Gia Nhu Nguyen et al. [28]	Blockchain with DBN (BC-DBN)	Improved security Identifies attacks accurately	Lacks scalability Less adaptable
Prabhat Kumar et al. [29]	Blockchain with BCoDSA-BiLSTM	Secure data transmission Healthcare data management	Computationally intensive Not applicable for large-scale networks
Nirmala Devi Kathamuthu et al. [30]	Deep Q-learning-based neural network (DQbNN)	Reduced cost Lower communication error	Cannot identify and eliminate emerging or unknown cyberthreats
Monire Norouzi et al. [31]	Genetic algorithm with random forest (GA-RF)	Safe and secure data transmission Predicts cyberattacks with high accuracy	Lacks generalizability High computational complexity

**Table 2:** Analysis of performance against different attacks.

Attack Type	Data Confidentiality Rate (%)	Throughput (%)	Processing/Running Time (ms)
DoS	97.70	95.70	4.10
DDoS	97.55	95.20	4.14
Brute Force Attack	98.10	95.80	4.05
Man-in-the-Middle Attack	97.45	94.95	4.2
Before attack (BA)	98.25	96.45	4

**Table 3:** ET comparison with the proposed and existing models.

File Size (kb)	ET (ms)			
	DSSA	SDT	DPSS	Proposed
500	0.07	0.67	0.19	0.014
1000	0.075	0.69	0.23	0.016
1500	0.079	0.75	0.26	0.019
2000	0.08	0.79	0.29	0.02
2500	0.082	0.85	0.45	0.021

**Table 4:** Resource utilization comparison with the proposed and existing models.

File Size (kb)	Resource Utilization (%)			
	DSSA	SDT	DPSS	Proposed
500	70.2	65	75.67	97.23
1000	70.56	67	78	97.67
1500	70.98	67.45	78.23	97.99
2000	71	67.67	78.87	98.06
2500	71.21	67.9	79.03	98.45

**Table 5:** Throughput comparison with the proposed and existing models.

File Size (kb)	Throughput (%)			
	DSSA	SDT	DPSS	Proposed
500	70.67	69	43.6	96.78
1000	85.9	70	43.67	96.9
1500	86	72	43.78	97.21
2000	86.67	75.9	44	97.56
2500	86.89	76	44.31	97.86

**Table 6:** DT comparison.

File Size (kb)	DT (ms)			
	DSSA	SDT	DPSS	Proposed
500	1.34	5.6	4.8	0.01
1000	1.56	5.67	4.98	0.013
1500	1.87	5.78	5	0.014
2000	1.99	5.90	5.45	0.016
2500	2.78	6.03	5.78	0.018

**Table 7:** RT comparison.

Sl. No	Technique	Running Time (ms)
1	DSSA	40
2	SDT	25
3	DPSS	20
4	Proposed	4

**Table 8:** Efficiency comparison.

Sl. No	Technique	Efficiency (%)
1	DSSA	80
2	SDT	75
3	DPSS	64
4	Proposed	93.45

**Table 9:** Confidentiality comparison.

File Size (kb)	Confidentiality (%)			
	DSSA	SDT	DPSS	Proposed
500	56	67	45	98.25
1000	56.5	68	45.78	98.4
1500	56.9	69	45.8	98.49
2000	57	70	47.9	98.6
2500	57.8	70.8	48	98.98

**Table 10:** ET comparison.

File Size (kb)	ET (ms)					
	PSObDNN	BC-DBN	BCoDSA-BiLSTM	DQbNN	GA-RF	Proposed
500	0.05	0.19	0.31	0.20	0.10	0.014
1000	0.062	0.23	0.38	0.24	0.17	0.016
1500	0.071	0.25	0.43	0.28	0.24	0.019
2000	0.077	0.29	0.49	0.32	0.30	0.02
2500	0.080	0.35	0.55	0.35	0.36	0.021

**Table 11:** DT comparison.

File Size (kb)	DT (ms)					
	PSObDNN	BC-DBN	BCoDSA-BiLSTM	DQbNN	GA-RF	Proposed
500	0.04	0.17	0.29	0.19	0.12	0.01
1000	0.058	0.22	0.34	0.23	0.19	0.013
1500	0.067	0.26	0.41	0.27	0.25	0.014
2000	0.075	0.30	0.48	0.31	0.32	0.016
2500	0.079	0.34	0.53	0.37	0.37	0.018

**Table 12:** Resource utilization comparison.

File Size (kb)	Resource Utilization (%)					
	PSObDNN	BC-DBN	BCoDSA-BiLSTM	DQbNN	GA-RF	Proposed
500	87.35	82.40	76.43	78.11	74.56	97.23
1000	87.5	82.59	76.51	78.2	74.77	97.67
1500	87.54	82.73	76.65	78.34	74.86	97.99
2000	87.89	82.85	76.71	78.58	74.97	98.06
2500	87.95	83.02	76.77	78.86	75.04	98.45

**Table 13:** Throughput comparison.

File Size (kb)	Throughput (%)					
	PSObDNN	BC-DBN	BCoDSA-BiLSTM	DQbNN	GA-RF	Proposed
500	85.05	80.81	74.33	76.64	70.23	96.78
1000	85.14	80.92	74.49	76.87	70.50	96.9
1500	85.22	81.06	74.71	76.98	70.72	97.21
2000	85.38	81.17	74.89	77.20	70.96	97.56
2500	85.75	81.26	75.02	77.47	71.08	97.86

**Table 14:** Running time comparison with hybrid models.

Sl. No	Hybrid Technique	Running Time (ms)
1	PSObDNN	17
2	BC-DBN	20
3	BCoDSA-BiLSTM	34
4	DQbNN	27
5	GA-RF	38
6	Proposed	4

**Table 15:** Data confidentiality rate comparison.

File Size (kb)	Data Confidentiality Rate (%)					
	PSObDNN	BC-DBN	BCoDSA-BiLSTM	DQbNN	GA-RF	Proposed
500	86	83.09	77.20	76.35	73.80	98.25
1000	86.4	83.25	77.34	76.43	74.01	98.4
1500	86.7	83.68	77.58	76.72	74.24	98.49
2000	87.15	83.91	77.92	76.85	74.39	98.6
2500	87.42	84.10	78.21	77.03	74.74	98.98

**Table 16:** System efficiency comparison with hybrid models.

SL. NO	Hybrid Technique	System Efficiency (%)
1	PSObDNN	89.32
2	BC-DBN	85.10
3	BCoDSA-BiLSTM	86.43
4	DQbNN	85.09
5	GA-RF	79.56
6	Proposed	93.45

**Table 17:** Performance assessment.

Performance	Proposed (AdaBoost SVM-Based GWO-IDEA)
Encryption time	0.014 (ms)
Throughput	96.45 (%)
Decryption time	0.01 (ms)
Running time	4 (ms)
Confidentiality	98.25 (%)
Resource utilization	97.23 (%)
Efficiency	93.45 (%)

## Data Availability

Data are contained within the article.
