# Correction: Abushark et al. Optimized Adaboost Support Vector Machine-Based Encryption for Securing IoT-Cloud Healthcare Data. *Sensors* 2025, *25*, 731

**DOI:** 10.3390/s26113433

**Published:** 2026-05-29

**Authors:** Yoosef B. Abushark, Shabbir Hassan, Asif Irshad Khan

**Affiliations:** 1 Department of Computer Science, Faculty of Computing and Information Technology, King Abdulaziz University, Jeddah 21589, Saudi Arabia; yabusharkh@kau.edu.sa; 2 Department of Computer Science, Aligarh Muslim University, Aligarh 202001, Uttar Pradesh, India; shassan.cs@amu.ac.in

In the original publication [1], we incorrectly cited a retracted reference “Karuppiah; Velrani, S.; Gurunathan, G. Secured storage and disease prediction of E-health data in cloud. *J. Ambient Intell. Humaniz. Comput.* **2021**, *12*, 6295–6306”. This reference has now been removed from the References.

**Text Correction**

In Section 5.3, the sentence “Consequently, the data sharing and secure authentication (DSSA) technique [42], secure data transmission (SDT) model [43], and disease prediction and secure storage (DPSS) [44] technique were validated with a designed method using metrics, such as confidentiality, running time, and resource utilization.” has been updated to “Consequently, the data sharing and secure authentication (DSSA) technique [42], and secure data transmission (SDT) model [43] were validated with a designed method using metrics, such as confidentiality, running time, and resource utilization.”.

The authors state that the scientific conclusions are unaffected. This correction was approved by the Academic Editor. The original publication has also been updated.

## References

[1] Abushark Y.B., Hassan S., Khan A.I. (2025). Optimized Adaboost Support Vector Machine-Based Encryption for Securing IoT-Cloud Healthcare Data. Sensors.

